# Poly[μ-azido-(μ_3_-nicotinato *N*-oxide)zinc(II)]

**DOI:** 10.1107/S1600536808039275

**Published:** 2008-11-29

**Authors:** Chun-Wei Xin, Fu-Chen Liu

**Affiliations:** a School of Chemistry and Chemical, Engineering, Tianjin University of Technology, Tianjin 300191, People’s Republic of China

## Abstract

The title compound, [Zn(C_6_H_4_NO_3_)(N_3_)], has been prepared by the reaction of nicotinate *N*-oxide acid, zinc(II) nitrate and sodium azide. The Zn atom is five coordinated by two azide anions and three nicotinate *N*-oxide ligands. Each nicotinate *N*-oxide bridges three Zn atoms, whereas the azide bridges two Zn atoms, resulting in the formation of a two-dimensional metal–organic polymer developing parallel to (100).

## Related literature

For background to metal–azide complexes, see: Escuer *et al.* (1997 ▶); Liu *et al.* (2005 ▶); Monfort *et al.* (2000 ▶); Shen *et al.* (2000 ▶).
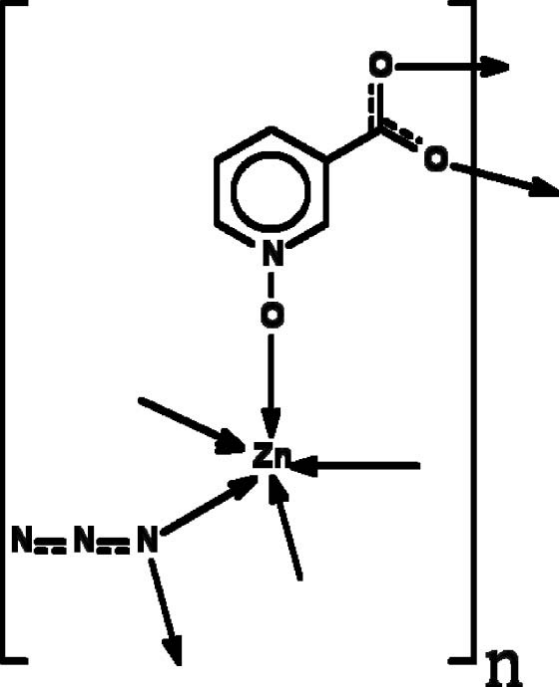


## Experimental

### 

#### Crystal data

[Zn(C_6_H_4_NO_3_)(N_3_)]
*M* *_r_* = 245.50
Monoclinic, 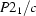

*a* = 8.1132 (16) Å
*b* = 6.1342 (12) Å
*c* = 15.786 (3) Å
β = 101.19 (3)°
*V* = 770.7 (3) Å^3^
*Z* = 4
Mo *K*α radiation
μ = 3.17 mm^−1^
*T* = 293 (2) K
0.20 × 0.18 × 0.15 mm

#### Data collection

Rigaku SCXmini diffractometer
Absorption correction: multi-scan (*ABSCOR*; Higashi, 1995 ▶) *T*_min_ = 0.786, *T*_max_ = 1.000 (expected range = 0.489–0.622)
7629 measured reflections
1761 independent reflections
1293 reflections with *I* > 2σ(*I*)
*R*_int_ = 0.091

#### Refinement

*R*[*F* ^2^ > 2σ(*F* ^2^)] = 0.065
*wR*(*F* ^2^) = 0.122
*S* = 1.12
1761 reflections
127 parameters
H-atom parameters constrained
Δρ_max_ = 0.50 e Å^−3^
Δρ_min_ = −0.52 e Å^−3^

### 

Data collection: *CrystalClear* (Rigaku, 2007 ▶); cell refinement: *CrystalClear*; data reduction: *CrystalClear*; program(s) used to solve structure: *SHELXS97* (Sheldrick, 2008 ▶); program(s) used to refine structure: *SHELXL97* (Sheldrick, 2008 ▶); molecular graphics: *ORTEPIII* (Burnett & Johnson, 1996 ▶) and *PLATON* (Spek, 2003 ▶); software used to prepare material for publication: *SHELXTL* (Sheldrick, 2008 ▶).

## Supplementary Material

Crystal structure: contains datablocks global, I. DOI: 10.1107/S1600536808039275/dn2407sup1.cif

Structure factors: contains datablocks I. DOI: 10.1107/S1600536808039275/dn2407Isup2.hkl

Additional supplementary materials:  crystallographic information; 3D view; checkCIF report

## Figures and Tables

**Fig. 1.**
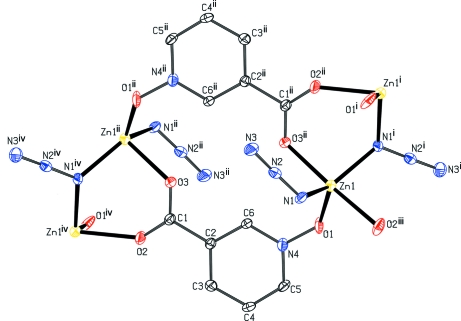
A partial view of the title compound showing the coordination of Zn atom with the atom-labelling scheme. Ellipsoids are drawn at the 30% probability level. H atom have been omitted for clarity. [ Symmetry codes: (i) -x+2, y+1/2, -z+3/2; (ii) -x+2, -y+2, -z+2; (iii) x, -y+3/2, z-1/2; (iv) x, -y+3/2, z+1/2]

## supplementary crystallographic information

### Comment

Metal azide complexes have attracted great attention in recent years. The azide anion have rich coordinated mode. (Shen,*et al.*, 2000). In this sense, several 1-D,2-D, and 3-D metal-azide complexes have been reported.(Monfort,*et al.*, 2000). In most of the compounds reported to date, the coligands are neutral organic ligands, while charged ligands are very scarce (Escuer *et al.*, 1997). Synthesizing high-dimensional compounds with azide and negatively charged ligands represents then a challenge for researchers working in this field.(Liu,*et al.*, 2005)

In the title compound, the zinc atom is five coordinated by two azide anions and three nicotinate N-oxide ligands (Fig. 1). Each nicotinate N-oxide bridges three zinc atoms whereas the azide is bridging two zinc atoms resulting in the formation of a two dimensional metal organic polymer developping parallel to the (1 0 0) plane.

### Experimental

A mixture of zinc(II)nitrate and sodium azide (1 mmol), nicotinate N-oxide acid(0.5 mmol), in 10 ml of water was sealed in a Teflon-lined stainless-steel Parr bomb that was heated at 363 K for 48 h. Pink crystals of the title complex were collected after the bomb was allowed to cool to room temperature.Yield 30% based on zinc(II). Caution:Metal azides may be explosive. Although we have met no problems in this work, only a small amount of them should be prepared and handled with great caution.

### Refinement

Hydrogen atoms were included in calculated positions and treated as riding on their parent C atoms with C—H = 0.93Å and *U*_iso_(H) = 1.2*U*_eq_(C).

### Crystal data

**Table:**

[Zn(C_6_H_4_NO_3_)(N_3_)]	*F*_000_ = 488
*M**_r_* = 245.50	*D*_x_ = 2.116 Mg m^−^^3^
Monoclinic, *P*2_1_/*c*	Mo *K*α radiation λ = 0.71073 Å
Hall symbol: -P 2ybc	Cell parameters from 6677 reflections
*a* = 8.1132 (16) Å	θ = 3.1–27.6º
*b* = 6.1342 (12) Å	µ = 3.17 mm^−^^1^
*c* = 15.786 (3) Å	*T* = 293 (2) K
β = 101.19 (3)º	Block, pink
*V* = 770.7 (3) Å^3^	0.20 × 0.18 × 0.15 mm
*Z* = 4	

### Data collection

**Table:**

Rigaku SCXmini diffractometer	1761 independent reflections
Radiation source: fine-focus sealed tube	1293 reflections with *I* > 2σ(*I*)
Monochromator: graphite	*R*_int_ = 0.091
*T* = 293(2) K	θ_max_ = 27.5º
ω scans	θ_min_ = 3.3º
Absorption correction: multi-scan(ABSCOR; Higashi, 1995)	*h* = −10→10
*T*_min_ = 0.786, *T*_max_ = 1.000	*k* = −7→7
7629 measured reflections	*l* = −20→20

### Refinement

**Table:**

Refinement on *F*^2^	Secondary atom site location: difference Fourier map
Least-squares matrix: full	Hydrogen site location: inferred from neighbouring sites
*R*[*F*^2^ > 2σ(*F*^2^)] = 0.065	H-atom parameters constrained
*wR*(*F*^2^) = 0.122	*w* = 1/[σ^2^(*F*_o_^2^) + (0.0419*P*)^2^ + 1.4372*P*] where *P* = (*F*_o_^2^ + 2*F*_c_^2^)/3
*S* = 1.12	(Δ/σ)_max_ < 0.001
1761 reflections	Δρ_max_ = 0.50 e Å^−^^3^
127 parameters	Δρ_min_ = −0.52 e Å^−^^3^
Primary atom site location: structure-invariant direct methods	Extinction correction: none

### Special details

**Table:**

Geometry. All e.s.d.'s (except the e.s.d. in the dihedral angle between two l.s. planes) are estimated using the full covariance matrix. The cell e.s.d.'s are taken into account individually in the estimation of e.s.d.'s in distances, angles and torsion angles; correlations between e.s.d.'s in cell parameters are only used when they are defined by crystal symmetry. An approximate (isotropic) treatment of cell e.s.d.'s is used for estimating e.s.d.'s involving l.s. planes.
Refinement. Refinement of *F*^2^ against ALL reflections. The weighted *R*-factor *wR* and goodness of fit *S* are based on *F*^2^, conventional *R*-factors *R* are based on *F*, with *F* set to zero for negative *F*^2^. The threshold expression of *F*^2^ > σ(*F*^2^) is used only for calculating *R*-factors(gt) *etc*. and is not relevant to the choice of reflections for refinement. *R*-factors based on *F*^2^ are statistically about twice as large as those based on *F*, and *R*- factors based on ALL data will be even larger.

### Fractional atomic coordinates and isotropic or equivalent isotropic displacement parameters (Å^2^)

**Table:**

	*x*	*y*	*z*	*U*_iso_*/*U*_eq_	
Zn1	0.92049 (8)	1.07685 (11)	0.77114 (4)	0.0250 (2)	
N2	1.2498 (6)	0.9185 (8)	0.8332 (3)	0.0259 (10)	
O3	0.9850 (5)	0.8093 (6)	1.1049 (2)	0.0246 (9)	
O2	0.8412 (5)	0.5276 (7)	1.1413 (3)	0.0396 (11)	
O1	0.7173 (5)	0.9338 (8)	0.7978 (2)	0.0395 (11)	
N4	0.7150 (5)	0.7746 (8)	0.8558 (3)	0.0261 (11)	
C6	0.8023 (7)	0.7911 (9)	0.9367 (3)	0.0229 (12)	
H6A	0.8716	0.9110	0.9526	0.028*	
N1	1.1263 (6)	0.8822 (7)	0.7773 (3)	0.0268 (11)	
C5	0.6141 (6)	0.6031 (10)	0.8310 (3)	0.0263 (13)	
H5A	0.5543	0.5953	0.7745	0.032*	
C4	0.5987 (7)	0.4421 (10)	0.8872 (4)	0.0308 (14)	
H4A	0.5276	0.3247	0.8700	0.037*	
C2	0.7901 (7)	0.6329 (9)	0.9958 (3)	0.0208 (12)	
N3	1.3654 (6)	0.9564 (8)	0.8842 (3)	0.0361 (13)	
C3	0.6906 (7)	0.4538 (10)	0.9712 (4)	0.0299 (14)	
H3A	0.6847	0.3418	1.0103	0.036*	
C1	0.8807 (7)	0.6564 (9)	1.0894 (4)	0.0232 (12)	

### Atomic displacement parameters (Å^2^)

**Table:**

	*U*^11^	*U*^22^	*U*^33^	*U*^12^	*U*^13^	*U*^23^
Zn1	0.0290 (4)	0.0231 (4)	0.0213 (3)	0.0007 (3)	0.0006 (2)	0.0007 (3)
N2	0.030 (3)	0.019 (2)	0.030 (3)	−0.001 (2)	0.008 (2)	0.000 (2)
O3	0.028 (2)	0.024 (2)	0.021 (2)	−0.0054 (17)	0.0008 (16)	−0.0007 (17)
O2	0.047 (3)	0.046 (3)	0.021 (2)	−0.024 (2)	−0.0064 (18)	0.011 (2)
O1	0.032 (2)	0.055 (3)	0.027 (2)	−0.014 (2)	−0.0085 (17)	0.025 (2)
N4	0.024 (3)	0.033 (3)	0.021 (2)	−0.002 (2)	0.002 (2)	0.005 (2)
C6	0.026 (3)	0.023 (3)	0.019 (3)	−0.006 (2)	0.000 (2)	−0.002 (2)
N1	0.025 (3)	0.025 (3)	0.026 (3)	0.004 (2)	−0.006 (2)	−0.007 (2)
C5	0.022 (3)	0.035 (4)	0.020 (3)	−0.007 (3)	−0.002 (2)	−0.004 (3)
C4	0.035 (3)	0.027 (3)	0.027 (3)	−0.012 (3)	−0.001 (2)	−0.008 (3)
C2	0.023 (3)	0.023 (3)	0.018 (3)	0.001 (2)	0.004 (2)	−0.002 (2)
N3	0.029 (3)	0.036 (3)	0.039 (3)	−0.006 (2)	−0.004 (2)	−0.004 (3)
C3	0.039 (4)	0.026 (3)	0.024 (3)	−0.010 (3)	0.004 (3)	0.002 (3)
C1	0.025 (3)	0.024 (3)	0.020 (3)	0.002 (2)	0.002 (2)	−0.003 (2)

### Geometric parameters (Å, °)

**Table:**

Zn1—O1	1.983 (4)	N4—C5	1.344 (7)
Zn1—N1^i^	2.031 (5)	C6—C2	1.363 (7)
Zn1—N1	2.040 (4)	C6—H6A	0.9300
Zn1—O3^ii^	2.079 (4)	C5—C4	1.349 (8)
Zn1—O2^iii^	2.125 (4)	C5—H5A	0.9300
N2—N3	1.134 (6)	C4—C3	1.392 (7)
N2—N1	1.220 (6)	C4—H4A	0.9300
O3—C1	1.255 (6)	C2—C3	1.374 (8)
O2—C1	1.225 (7)	C2—C1	1.523 (7)
O1—N4	1.342 (6)	C3—H3A	0.9300
N4—C6	1.338 (6)		
			
O1—Zn1—N1^i^	112.69 (19)	C2—C6—H6A	119.9
O1—Zn1—N1	115.93 (19)	N2—N1—Zn1^v^	120.5 (4)
N1^i^—Zn1—N1	130.49 (12)	N2—N1—Zn1	118.5 (4)
O1—Zn1—O3^ii^	96.82 (16)	Zn1^v^—N1—Zn1	115.5 (2)
N1^i^—Zn1—O3^ii^	93.02 (17)	N4—C5—C4	120.7 (5)
N1—Zn1—O3^ii^	90.14 (16)	N4—C5—H5A	119.7
O1—Zn1—O2^iii^	87.86 (18)	C4—C5—H5A	119.7
N1^i^—Zn1—O2^iii^	85.13 (18)	C5—C4—C3	119.2 (5)
N1—Zn1—O2^iii^	87.80 (17)	C5—C4—H4A	120.4
O3^ii^—Zn1—O2^iii^	175.31 (17)	C3—C4—H4A	120.4
N3—N2—N1	178.4 (6)	C6—C2—C3	119.5 (5)
C1—O3—Zn1^ii^	123.1 (4)	C6—C2—C1	120.7 (5)
C1—O2—Zn1^iv^	140.4 (4)	C3—C2—C1	119.7 (5)
N4—O1—Zn1	126.1 (3)	C2—C3—C4	119.1 (5)
C6—N4—O1	121.4 (5)	C2—C3—H3A	120.4
C6—N4—C5	121.1 (5)	C4—C3—H3A	120.4
O1—N4—C5	117.3 (4)	O2—C1—O3	127.3 (5)
N4—C6—C2	120.3 (5)	O2—C1—C2	116.6 (5)
N4—C6—H6A	119.9	O3—C1—C2	116.1 (5)

Symmetry codes: (i) −*x*+2, *y*+1/2, −*z*+3/2; (ii) −*x*+2, −*y*+2, −*z*+2; (iii) *x*, −*y*+3/2, *z*−1/2; (iv) *x*, −*y*+3/2, *z*+1/2; (v) −*x*+2, *y*−1/2, −*z*+3/2.
